# Book Reviews

**Published:** 1808-05

**Authors:** 


### CRITICAL ANALYSIS OF THE RECENT PUBLICATIONS ON THE DIFFERENT BRANCHES OF PHYSIC, SURGERY, AND MEDICAL PHILOSOPHY.

*A Letter to the Right Honourable Sir Joseph Banks, P. R. S. on the Causes and Removal of the prevailing Discontents, Imperfections, and Abuses in Medicine, from* THOMAS BEDDOES, M. D.

IT is the great advantage of a popular government that "every thing is discussed." By the various statements of individuals, we at least arrive at a knowledge of the oppression which each feels or apprehends; and if we are prevented from applying remedies for one, lest we should injure another, we are also withheld from multiplying inconveniences where some must always exist. All our readers will rejoice, that the important subject of medical reform is taken up by the Author before us. His comprehensive mind, his extensive reading, his unreserved mode of expression, and his well known professional rank, lead us to expect impartiality and information. But there is another part of the history of Dr. Beddoes, which renders him peculiarly proper to engage in the present contest. Like Teresias of old, he may claim an intimate knowledge of the whole question, being actually *alumnus* of the Northern and the Southern Schools of Great Britain.

"Let me, therefore, (says Dr. B.) briefly state, that I went to Edinburgh as an Oxford Bachelor of Arts, passed there three winters and one summer, was perpetually at the lectures of the professors, and in the Societies of the Students. You may think it probable that I have no humiliating associations connected with Edinburgh, if I add that I can never hope to be of so much consequence among my equals any where else, since the students heaped upon me all those distinctions which you know it is in their power to confer. Few individuals, certainly, have ever had a better opportunity of knowing any school. I have seen other schools of medicine, conversed and corresponded much, from that time to the present, with pupils and professors, studied their methods, and the productions as well of the youth as of the seniors; so that I cannot accuse myself of having omitted any thing by which I might be enabled to form an opinion concerning this grand question of medical reform.

"After comparing, on the spot, the means with the end, I certainly did conceive that a more deliberate process would be preferable, and that a method of instruction, in some other respects, materially different, would form physicians far more trustworthy. This opinion, various members of the medical societies could, I dare dare say, testify that I expressed; and every thing that I have since seen of practice and of literature has tended to confirm it. After a lapse of years, and without the smallest communication, it is satisfactory to find the associated faculty and their correspondents concurring to make it the basis of a legislative measure, and certainly without being actuated by the least ill will towards any medical school in the universe. I know not whether any impartial person, after seriously reflecting upon the surest way of advancing in so difficult a study, ever surveyed the medical classes at Edinburgh. He would see that perpetual bodily hurry, which is generally attended with a good deal of confusion of mind. No sooner does the college hour bell toll, than the audience rush out in full stream, leaving the last word half finished in the mouth of one professor, not a few fearing lest they should miss the first words of another. Will you call this mere juvenile ardour? The young men there were generally, and doubtless still are, earnest in their pursuits; but it was a common feeling, that each attempted too much at once; and if it be true, that figures and hues which are to last, must be laid again and again on the mind, with pauses between to allow them to fix, somewhat as in fresco painting, this feeling would appear to be right. A calculation had been made, and the required attendance distributed as well as possible through the three years. Considering the number of professors, and the necessity for those, who were to trust to this school solely, to attend certain courses, (as the anatomical, practical, and clinical) two or three times; considering, besides, that the merit of *out-lecturers* will have claims upon the inquisitive, and that many had no other chance for acquiring a smattering of natural philosophy and natural history, how could any student, and especially the most ardent, avoid attempting too much at once? The consequence was too apparent. Our academical architects, in their hurry to finish the structure, failed to lay a solid foundation.

" But the contrary is, in effect, affirmed. The senate appeal to the advancement of medical science, by the graduates of their school. Here again, unless we had a measure for the quantity, and a test for the quality of improvement, which may be fairly expected in a given time, to what can we have recourse but the inconclusive expression of opinion? While some acquiesce or admire, others viewing the immense number of labourers sent from Edinburgh into the field of medicine, may feel astonished at the insignificance and small value of the produce.

" When persons, however rude and undertaught, have certain objects constantly under superintendance, they inevitably acquire some insight into their properties, and skill in their management. Were the Hindoos indebted to instruction in mathematics for their proficiency in astronomy? Of all knowledge, the beginnings, and of some the great part, are owing to the lessons of opportunity, which has forced improvements upon the faculties. The improvements in the steam engine are in part contrivances by children, eager eager to go on uninterruptedly with their play. In estimating the merits of any school, are we not, therefore, to subtract, as well as we can, the portion due to necessary natural proficiency from the whole mass of improvement made by the disciples ? And will not this be greater as they are more numerous ? Let us glance, however, at particulars.

"Fever and febrile disorders form incontestibly the largest and most important department within the sphere of the physician. No school, perhaps, has enjoyed greater celebrity for its doctrines concerning fever; and our age has certainly afforded too many opportunities of putting the proficiency of the pupils to the test. Yet when certain fevers, of which the character is to be traced in the records of medicine, from Hippocrates downwards, became, as is too well known, epidemic in various regions of the earth, during the last twenty years, I do not know that any men were more confounded or more helpless than the pupils of Edinburgh ; they had the fairest possible field for a display of skill, yet their practice was either more unsuccessful than that of the despised French schools, for example, or at least not more successful. And if any one should succeed in persuading you that the sick did not perish in greater multitudes under their hands, (which yet I hardly think they will accomplish, if you have the whole of the evidence before you) what argument would this afford against an attempt to improve the whole study of medicine, by providing that the students shall carry to the infected city or camp a larger stock of knowledge ? There stood, as I have said, on record, a good deal of that information which the occasion demanded. At college, however, for what was there time but attending to the oral instruction of the masters ? From the constitution of the place, I shall afterwards give my reasons for thinking why it was not likely that they should supply for themselves what the discipline they had undergone left deficient."

We have given this long extract, as the readiest means of introducing Dr. Beddoes to our readers. The passage next contains some just eulogia on the Navy and Army practitioners, who have been guided not by their professors but by actual observatiion. It is regretted, in the words of Lord Bacon, that neither the records of practice, as contained in medical books, nor the experience derived from empiricism, have enabled physicians to set down certain "experimental medicines for particular diseases." On this occasion, we are glad to see justice done to a writer, whom we accused Dr. Beddoes of passing over too slightly.* We are still better pleased that he calls the attention of Dr. Currie's friends to the improper mode of expression he has used on this occasion.

"It

* Dr. Jackson. See our Review of Dr. Beddoes's "Exercise on Fevers."

"It seems to me (says our Author) full as fortunate that Dr. Currie was originally destined to a different calling, and that he visited a distant part of the world with other views than those of a medical student, as that he studied at Edinburgh. Thus his faculties must have been enlarged and quickened; but even thus, has he escaped altogether the effects of a cramped medical education? That his notions concerning fever are much too limited, and so far dangerous, must, I think, appear to every one acquainted with the history, and especially the anatomical history, of the disease. Hence the rashness with which he has scattered the seeds of discredit over the results of a fellow-experimenter on fever; results confirmed, to say the least, as amply as his own, by prior, by contemporary, and subsequent experience. I should also be glad to learn from intelligent and impartial readers of Dr. Currie, if his mind do not seem frequently labouring, as under night-mare oppression, from a mass of indigestible hypothesis, laid upon it in the school of medical practice, which he frequented. The defect, if it exist, I should, in great measure, impute to a most amiable motive, *pietas erga preceptorem*; but if the preceptor, or the genius of place, had initiated him more extensively in the results of faithful experience, might he not have had the piety equally without the defect."

"Our documents relative to improvements in the treatment of disorders, not so general or alarming as fever, are necessarily less copious; but here, whether I advert to the still remaining imperfections of medicine, to the atchievements of the pupils of other schools, or to what may be reasonably hoped from seminaries better constituted, I can find little answering to the assumptions of the senate. I suspect that a good judge of medical stock would find many physicians educated during the greatest splendour of the Edinburgh school, whatever local admiration several may enjoy, among the sorriest sheep in the whole flock of Esculapius. Edinburgh graduates, in short, no less than others, have 'professed more than laboured, and yet laboured more than advanced,' our art."

The succeeding paragraphs dwell with much propriety on the absurd assertion of the Edinburgh Senate, that three years are sufficient to complete the studies of a physician. It is admitted with much candour, that neither Oxford nor Cambridge is, or ever was, considered as a proper seminary to form the practitioner. But are they not sources of that αληθιν παιδεια, by which men learn the right mode of studying every science? Are they not those schools, in which the connection between the various branches of instruction is well understood; in which the scholar hears not what the professor chooses to teach, but what is established on inferences founded on laws, which are admitted by the common consent of mankind? Thus prepared, can a youth be better fitted to improve by that vast assemblage of facts, by those continual instructions which he sees and hears at the bedside in a

London Hospital? Accordingly, our Author asserts, that small as the comparative number is, of English to Scotch graduates, still if we judge by the fruits, that is, by their respective publications, we shall find in the former all the advantage that we might expect from the maturity of time.

On the contemptuous manner in which the Edinburgh graduates view the diplomatists of Aberdeen and St. Andrews, our Author is particularly pleasant. On the whole, he seems to think that the latter, who rarely think of being *dubbed* till their practical success has given them some celebrity, may be better qualified than those raw graduates, who with "a mouthful but not with a bellyful," may persuade a knot of old ladies that they come from Scotland full charged with the healing art. But when to this we add the frequency with which a graduate is *ground* into responses, instead of being grounded in his art, the conclusion seems to be, that recommendations to St. Andrews, syllogisms at Oxford, and grinding at Edinburgh, stand pretty much on the same ground.

We have next an account of the several Universities on the continent, above most if not all of which, the preference appears due to Paris. But as Dr. Beddoes has given us a scheme of Education of his own, we shall transcribe it, in hopes that some of our Readers may be induced to favour us with their communications on this subject. To such, the outlines offered by an experienced and impartial practitioner will be very acceptable, and we doubt not that our Author will be well pleased with their corrections.

"You will hardly have had the patience to read thus far without expecting the positive, and, indeed, sole decisive proof of the hostility of the pretensions of the Edinburgh senate to the public welfare. This consists in the delineation of a course of study, from which it shall appear that a young man can employ five or six years at medical seminaries with advantages of the most essential kind, which he must forfeit on starting sooner as an independent practitioner, and which graduates of three years, as such, can therefore never possess. I beg leave, then, to submit the following scheme, of which I hope that the discerning and experienced will approve the principle. It is too much to expect coincidence in minutiæ.

"First year. *Dissection, anatomical lectures, reading, drawing, and comparison of anatomical engravings with the objects of nature.* For relaxation, as much as for instruction, *a course of chemistry and elementary reading;* — this for winter. In spring and summer, *a course of comparative anatomy, dissection of animals, botany, and physiological reading,* till the winter of the

"Second year. *Anatomy exactly as before, attendance on clinical lectures in surgery;* if none are given, *close study of surgical cases, particularly of surgical accidents at first morbid anatomy practically,* by every opportunity from this time forward. In the spring, summer, and autumn, *practical chemistry, pharmacy, botany, materia medica.*

"Third

"THIRD YEAR. In winter — *Anatomy and surgery* still; but external diseases now more than accidents. Spring, summer, and autumn — *Midwifery, medical jurisprudence, comparative anatomy, physiology,* and the other before-mentioned pursuits occasionally.

"FOURTH YEAR. *Anatomy to be kept up, lectures on the practice of medicine, clinical lectures. Observation of medical cases, and practical reading,* to be a chief occupation through this year. The student may pass it at Edinburgh, at least from October to July.

"FIFTH AND SIXTH YEARS. *Close attendance on hospitals, with practical reading and lectures,* at Paris and Vienna if accessible; otherwise in London. During the autumn of this or the preceding year, some time, if possible, to be employed in attending military hospitals, especially in the field.

"During the summers, *oral instruction, as it best offers, in other branches of natural history besides botany, in natural philosophy, and in the speculative sciences,* if in these last any lectures should promise more than books. From one or the other, *the acquisition of as many facts as possible concerning the mental operations,* should be considered as an essential part of the stock of the knowledge necessary to the physician."

The only objection we see to this plan is, that the student is not required to pay sufficient attention to the practice of teachers during this long period. In our opinion this, from the beginning, should form a part of his employments. The summers also should be devoted to lectures on the theory and practice of physic, but not till the student is sufficiently informed to judge in some measure for himself, as well to apply what he hears to what he has seen.

The Author dwells much on the importance of visceral anatomy, and also of morbid anatomy. Perhaps, he expects too much from the latter; but in a science so complicated, can we doubt the necessity of every means of information? The name of Mr. Hunter is very properly introduced, to prove, by a convincing illustration, how much we are mistaken if we suspect that accuracy in the common organs of sense is inconsistent with refined speculation. The contrary appears to be our Author's opinion; and we admit with him, that "contemplation cannot find a stage so advantageous from which to soar; that those who cannot reason upon a stock of such ideas, would never otherwise be able to reason to any purpose; and that this road is still the best, even for those who are incapable of going so fast or so far." But we may be allowed to remark, that however desirable the association of accurate sensual organs with strong powers of ratiocination may be, we cannot often expect to see them united, as they were in Mr. Hunter. We conceive also, that no one will dispute the advantage derived from such an union, so that we are not quite prepared to determine the precise intention of our Author in this passage. But it seems still more remarkable, that between this and a subsequent passage, in which Mr. Hunter is introduced, the advantages of erudition should be exemplified in the late Dr. Hebberden. Perhaps it was intended to show, that Edinburgh could neither boast the learning of Oxford or Cambridge, nor the practical advantages of London.

"To show the general necessity of erudition to observation and improvement, it may be sufficient to refer to the progress of knowledge, in which we perpetually see one man advancing further because another had advanced so far. Allow me, however, to advert to a single example. I will choose, as the fairest, that of Hebberden; in whose works *nihil e scriniis aliorum compilatum videmus;* and in whom, *summa est fides, summa auctoritas, pura, incorrupta, intaminata.* But, Sir, was Hebberden pure, somewhat after the pattern of those children of nature, the imaginary savages of Rousseau, because he lived a stranger to the corruptions of art? Was he original, because never in the way to be prepossessed? Nothing like it. He was singularly learned—*eruditionis omnis amantissimus, ipse omni eruditione ornatus,* with that of his own profession among the rest. He did not avoid the snares of hypothesis, because he never approached them, but because he saw where and how they were laid. *Ab eo, quicquid vel hilum saperet Theoreticam artem . . . . . . . quam maxime abhorrens.* And what instruction, I beseech you, is so effectual as that of bad examples taken right?"

"I rejoice at having only one Edinburgh objection more to notice, (though if I knew of any better arguments to the same purport, I should hold their production indispensable.) In noticing this, I shall be led to a consideration equally important with the preceding. The committee of association, as we have seen, propose to put "respectable schools of physic" on the same footing with universities, as to the qualifying effect of residence. The senate, and you cannot be surprised at it, take fire at the proposal. To admit "certificates from *schools of physic* may," they object, "prevent the possibility of ascertaining a regular education; schools may be self-erected, and subject to no controul." The omission of a word in this objection does, to be sure, make some little change in the sense. "*Respectable* schools" are specified.—The senate run on arguing, as if it had stood "any schools whatever." The paper, indeed, wanders away too far into minutiæ of regulation, which are not yet before the public, and do not belong to the discussion of the principle.

"The provisions might have been exposed to just censure. Respectable schools are not easily distinguishable in terms from the reverse, though they will distinguish themselves, and none others be frequented, unless they have the power of conferring degrees. But to oppose conferring equality of right upon schools which have displayed equal power with any university in qualifying for practice, would not sound quite so liberal as the conditional offer to surrender a lucrative privilege.

"You

"You might, I suspect, find among the medical men around you, some who would go so far as to ascribe, to the instructions of the late Mr. Hunter alone, greater effect in forming the practitioner, than to those of all the Edinburgh teachers of his time put together. A majority of the medical world would place him far above the best singly; and does not the whole face of medicine, if we compare the impression left upon it by the one and the other, corroborate the preference? Along with Mr. Hunter, the student might have attended his brother, who was the best modern teacher of anatomy, Dr. G. Fordyce, and others, together with the hospitals. Was it just that advantages, so important to the essence, should go no way towards the form of a Doctor in Medicine? Had we only to decide, whether the larger share of profit from medical education should go to London or Edinburgh, I would either not waste a dozen drops of ink on the question, or vote for Edinburgh as being in possession. But superior interests are at stake. And now, what is there at Edinburgh in the shape of instruction that London need view with despair? In what respect ought we to deem a London education (equal in time and diligence) with a St. Andrew's diploma, inferior to an Edinburgh education and diploma? In what but the interposition of the grinders, those *dii ex machina* in the great scene of Edinburgh Doctor dubbing."

It is next remarked, that London, like the metropolis of other countries, should have the advantage of an university; perhaps it would be enough if the London College had the power of giving degrees as well as licences. Probably, however, the latter may be sufficient, for if Fellows of the London College succeed better than Licentiates, it arises more from the knowledge the wealthy part of the public has acquired of their talents at the English Universities, than from any value in which their titles are held.

We have dwelt so long on the early part of this pamphlet, because it contains almost the whole of Dr. Beddoes's strong arguments. The remaining pages are replete with point, and often with humour, and serve to show, in a still stronger view, the absurdity of that arrogance which the Edinburgh school assumes. The whole tends to prove, what every honest practitioner must admit, that for a practical art, practice only can prepare the artist; that whatever assistance a student may derive from his teacher, must be, as in every science which unites demonstration and reasoning, only by teaching him how to apply what he sees before him. We may perhaps be accused of partiality when we urge, that the English Universities and London hold out the most ample means for real genius and industry to work upon; and that though dullness may learn the language and grimace of Physic, she will never form the useful practitioner in any cases which require precision. ——But, to return to Dr. Beddoes. This we shall do, only to thank him for the amusement he has afforded us, the information we have received from him, and to recommend his work to the perusal of our Readers.

*Observations on the Utility and Administration of Purgative Medicines in several Diseases.* By JAMES HAMILTON, M. D. Fellow of the Royal College of Physicians; and of the Royal Society of Edinburgh; and Senior Physician to the Royal Infirmary of that City. Second Edition, corrected and enlarged.

WE have already paid our tribute to the merits of this author when the first edition was published. We must, however, again remark, that the necessity of many of the observations must have been greater in Edinburgh than in London, as we have never seen among our best practitioners, that great apprehension of cathartic remedies at which Dr. Hamilton more than hints.

Besides a few alterations of less importance, the principal additions to this edition consist of a Chapter on Hysteria, and another on Tetanus. They are certainly not without their use; but whoever reflects on the uncertainty of the period, and returns in the paroxysms of Hysteria, will doubt, whether the cases related in the Appendix were much assisted by the remedies prescribed. As to Tetanus, we could almost have wished the Chapter had been omitted, or that the remarks had been more general, and written in such a manner only as to direct the attention of practitioners to the probable advantage they might derive from purgatives by analogy with other diseases. The author cannot mean to produce these cases as resembling those dreadful instances of Lock-jaw, which occur from slight or severer wounds, and which have hitherto baffled every attempt at cure. It is well known, that even in the worst cases, if the patient survives the seventh day, the case usually becomes chronic; and the event, though tedious, is for the most part favorable. Two of the cases here related, were probably as Dr. H. suspects, sympathetic of some diseased action, or want of action, in the intestines; the others were chronic complaints, and though, doubtless, treated with much judgment, could have no analogy whatever with acute Tetanus.

It does much credit to the author of so popular a work, that he has published in a separate pamphlet whatever is new in the present edition, for the convenience of those in possession of the former.

